# Improving analytical methods for protein-protein interaction through implementation of chemically inducible dimerization

**DOI:** 10.1038/srep27766

**Published:** 2016-06-10

**Authors:** Tonni Grube Andersen, Sebastian J. Nintemann, Magdalena Marek, Barbara A. Halkier, Alexander Schulz, Meike Burow

**Affiliations:** 1 Center for Dynamic Molecular Interactions (DynaMo), Department of Plant and Environmental Sciences, University of Copenhagen, Thorvaldsensvej 40, 1871 Frederiksberg C, Denmark; 2 Department of Plant and Environmental Sciences, Faculty of Science, University of Copenhagen, Thorvaldsensvej 40, 1871 Frederiksberg C, Denmark

## Abstract

When investigating interactions between two proteins with complementary reporter tags in yeast two-hybrid or split GFP assays, it remains troublesome to discriminate true- from false-negative results and challenging to compare the level of interaction across experiments. This leads to decreased sensitivity and renders analysis of weak or transient interactions difficult to perform. In this work, we describe the development of reporters that can be chemically induced to dimerize independently of the investigated interactions and thus alleviate these issues. We incorporated our reporters into the widely used split ubiquitin-, bimolecular fluorescence complementation (BiFC)- and Förster resonance energy transfer (FRET)- based methods and investigated different protein-protein interactions in yeast and plants. We demonstrate the functionality of this concept by the analysis of weakly interacting proteins from specialized metabolism in the model plant *Arabidopsis thaliana*. Our results illustrate that chemically induced dimerization can function as a built-in control for split-based systems that is easily implemented and allows for direct evaluation of functionality.

Over the past decades, it has become apparent that protein-protein interactions (PPIs) play a vital role in posttranslational regulation of virtually all biological systems. Meta-data analyses show that changes in PPIs can lead to incorrect subcellular organization that might underlie severe medical conditions such as Alzheimer’s and other genetic diseases^1^,^2^. While entire interactomes for a range of model systems have been investigated using high-throughput analyses, PPIs that are of dynamic nature remain underrepresented as they are hard to distinguish from negatives^3^. One example of a system that is regulated by highly elusive PPIs is the production of purines. The biosynthetic machinery responsible for purine synthesis assembles only upon purine starvation, as the individual enzymes require specific co-factors for interaction^4^.

Within plants, intracellular coordination of multi-enzymatic pathways is especially important as they produce a plethora of specialized compounds that are essential for survival. These pathways are tightly regulated and evidence suggesting that PPIs play an important role in their subcellular organization is emerging^5^,^6^. The biosynthesis of the defensive metabolites glucosinolates (GLS) in the model plant *Arabidopsis thaliana* (hereafter Arabidopsis) is one of the best-characterized specialized pathways to date, and therefore represents a good model to study the occurrence of PPI within specialized plant metabolism. GLS are synthesized at the ER-cytosol interface^7^, where the pen- and ultimate reactions are catalysed by cytosolic UDP-dependent glucosyltransferases (UGTs) of the UGT74 subclass^8^,^9^ and specific sulfotransferases (SOTs)^10^ respectively. UGT and SOT activity has been found to co-purify only under certain conditions^11^,^12^,^13^, suggesting that theses enzymes might interact weakly.

To analyse PPI between two proteins of interest a number of methods are available. Arguably, the most widespread approaches are based on PPI-dependent reconstitution of a split protein, which upon PPI-dependent reassembly leads to a measurable endpoint signal^14^. Systems that apply this principle include two-hybrid^15^- and split ubiquitin-based analyses in yeast or mammalian cells^16^,^17^,^18^. Moreover, in such strategies, bimolecular fluorescence complementation- (BiFC)^19^ and/or Förster resonance energy transfer-based (FRET) analyses^20^ are often used for validation of PPIs^14^. Although forming the basis for our knowledge on the occurrence of PPIs, the principle behind all complementary and split reporter methods comes with some disadvantages. For analysis of weak and dynamic interactions, methods applying split and dual reporter systems lack a built-in positive control, rendering positive readouts difficult to compare across experiments and platforms. Additionally, upon misfolding, mistargeting, or low expression of the investigated protein(s), a false negative readout of a split-based system cannot be directly distinguished from a true-negative. The ability to conditionally force the split reporter to reassemble independently of the investigated proteins would serve as an elegant way to overcome these issues and further increase the applicability of this otherwise highly successful concept.

Intriguingly, the 12 kDa human FK506 binding protein (FKBP12) and the 100-amino acid domain of the kinase Target of Rapamycin (TOR) known as the FKBP-rapamycin binding domain (FRB) do not directly interact, but dimerize in the presence of the chemical rapamycin^21^. This interaction is well characterized and has been exploited for chemically induced activation of e.g. signalling cascades, changes in subcellular localization and regulation of protein stability in mammalian and yeast systems^22^,^23^. Due to the inert nature and small size of FKRB12 and the FRB domain, they represent ideal candidates for incorporation into complementary reporter tag systems in order to overcome the abovementioned issues.

In this work, we investigate if the chemically induced dimerization (CID) between FKBP12 and the FRB domain can be used as an internal control to improve systems that are based on the ‘spilt’ reporter concept. We implement FKBP12 and FRB into the reporters of split ubiquitin-, BiFC- and FRET-based PPI systems and demonstrate their functionality by evaluating the interaction between specific UGTs and SOTs from the Arabidopsis GLS biosynthetic pathway. In combination, our data illustrate that the addition of CID into split reporter systems can serve as a built-in positive control. This implementation thereby not only improves the workflow required for a thorough evaluation of PPI between two proteins of interest, but also enhances the efficacy and applicability of an already well-established and highly successful concept.

## Results

### FKBP12 and the FRB domain are functional as chemically induced dimers in a split ubiquitin system

**Figure 1:**
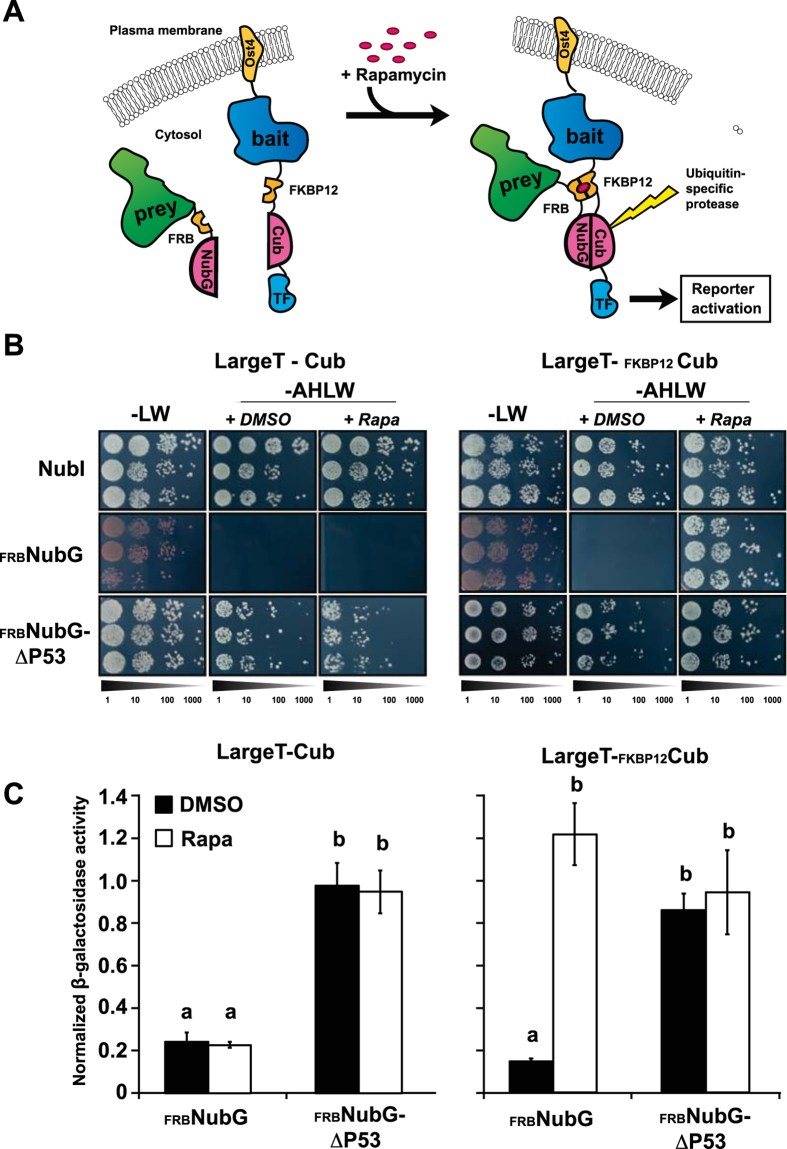
Evaluation of functionality of chemically induced interactions in a yeast-based split ubiquitin system. (**A**) Upon negative interaction between two proteins of interest (prey and bait), addition of rapamycin to the media will lead to an induced interaction between FKBP12 and the FRB domain incorporated into the bait and prey reporters. This leads to a bait- and prey-independent reassembly of the split ubiquitin and activation of the downstream reporters. (**B**) Growth assay of LargeT-Cub baits with and without FKBP12 in combination with NubI (positive control) as well as an FRB-containing NubG prey vector alone or including its interaction partner ΔP53. Cells from three independent transformations were grown in a dilution series from OD_600_ = 0.5 to 0.5:1000 on synthetic dropout media without leucine and tryptophan (−LW) as well as media selective for interacting bait-prey combinations (−AHLW). Inducibility of interactions was assessed by adding DMSO (+ DMSO) or 10 μg/mL rapamycin (+ Rapa) to the -AHLW media. (**C**) β-galactosidase assays to address the relative interaction strength of the protein pairs. The β-galactosidase activity was normalized to that of NubI-containing bait combinations. Different letters indicate significant differences between groups (One-way ANOVA, P < 0.01, N = 8).

The yeast (*Saccharomyces cerevisiae*) platform represents the most successfully applied approach to analyse PPI^14^. Therefore, we initially adapted the human FKBP12 and the FRB domain to be applied in the versatile Dualhunter^TM^ split ubiquitin system that allows analysis of both membrane-anchored and cytosolic proteins (Fig. 1A) (Dualsystems Biotech). The employed yeast strain (NMY51) was mutated to carry specific mutations in the TOR1 gene (*TOR1-1)* as well as a knockout of the protein FPR1 (Δ*fpr1)*, which in combination confer resistance to rapamycin^24^ (supplementary Fig. S1). In split ubiquitin-based systems, functionality of the bait is evaluated by co-expression with the non-modified N-terminal part of ubiquitin (NubI) that has high affinity towards the C-terminal part of ubiquitin (Cub). The bait construct therefore consist of the protein of interest followed by Cub and a LexA-VP16 artificial transcription factor, which is released upon ubiquitin reconstitution, in order to activate the end-point reporters^18^ (Fig. 1A). To make the reconstitution of ubiquitin PPI-dependent, a modified non-interacting version (NubG) is fused to the prey^16^. To create a CID version of this system we incorporated FKBP12 into the N-terminus of the bait tag, while the FRB domain was fused to the N-terminal part of NubG in the prey, in a similar manner (Fig. 1A).

Upon co-transformation of the FKBP12- and FRB domain-containing bait and prey constructs in the rapamycin-resistant yeast strain, growth was assayed on PPI selective media (−AHLW). Expression of the FKBP12- and FRB-fused Cub and NubG constructs alone was found to activate the reporters solely in the presence of rapamycin (Fig. 1). This demonstrates that the CID domains are functional in the modified NMY51 yeast strain and that they do not self-interact. To evaluate whether the modified reporters are applicable in combination with proteins of interest, we employed the SV40 antigen LargeT and the protein ΔP53 as bait and prey, respectively. These proteins have a high affinity towards each other^25^ and are employed as controls in the Dualhunter^TM^ split ubiquitin system. Independent of the presence of rapamycin, we observed a strong activation of the reporters when LargeT bait fusions (with or without the FKBP12) were tested in combination with NubI alone, or a ΔP53 prey construct containing FRB (Fig. 1B,C, supplementary Fig. S2). However, only when FKBP12 was present in the LargeT bait, the combination with FRB-NubG gave rise to activation on selective media containing rapamycin (Fig. 1B,C). No activation was observed between the FKBP12-containing LargeT bait and NubG alone in the presence of rapamycin (supplementary Fig. S2). Thus, the interaction between the FKBP12-LargeT-Cub and ΔP53-FRB-NubG was not affected by the presence of rapamycin, indicating that the CID tags do not interfere with bait and prey interactions.

### CID split ubiquitin for evaluation of relative interaction strengths?

**Figure 2:**
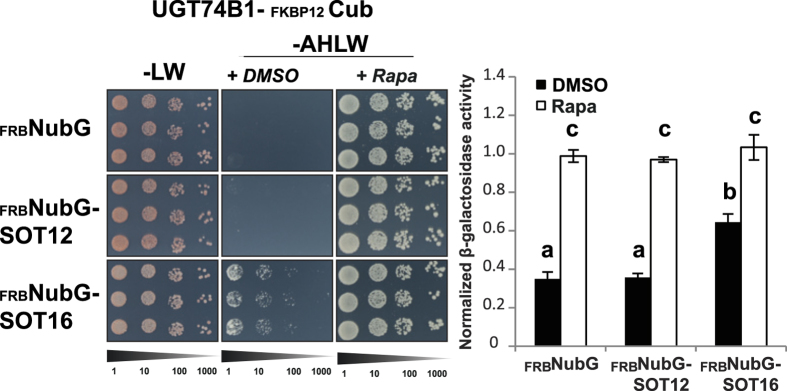
Evaluation of chemically induced interactions between UGT74B1 and two sulfotransferases from Arabidopsis. Growth assay of a chemically inducible UGT74B1-Cub bait in combination with FRB-NubG (negative control prey) or two prey protein fusions containing SOT12 or SOT16. Cells from three independent transformations were grown in a dilution series from OD_600_ = 0.5 to 0.5:1000 on synthetic dropout media without leucine and tryptophan (−LW) as well as media selective for interacting bait-prey combinations (−AHLW). Inducibility of interactions was addressed by adding DMSO (+DMSO) or 10 μg/mL rapamycin (+Rapa) to the −AHLW media. The β-galactosidase activity was normalized to that of NubI-containing bait combinations. Different letters indicate significant differences between groups (One-way ANOVA, P < 0.01, N = 8).

With functionality of CID in a split ubiquitin setup confirmed, we set out to investigate if this modified system can be employed to study relative interaction strengths. We employed the proteins UGT74B1 and SOT16 from the Arabidopsis GLS pathway as bait and prey respectively. To evaluate specificity, we included the closely related SOT12, which functions in the synthesis of brassinosteroids^26^ as well as detoxification of xenobiotics^27^. Co-transformation of FRB-NubG alone and the UGT74B1-containg bait construct did not give rise to activation of the reporter, demonstrating that this bait did not auto-activate the reporters under the applied conditions (Fig. 2). A similar observation was made in combination with the SOT12-FRB-NubG prey construct (Fig. 2). In both cases, strong activation of the reporters could be observed upon addition of rapamycin to the media, illustrating that the UGT74B1 and SOT12 constructs were indeed functional in the split ubiquitin system and that the readout represented a true-negative interaction.

In the case of the combination of the UGT74B1 bait and the SOT16 CID prey construct, we observed reporter activation when grown without addition of rapamycin which could be further increased by addition of rapamycin to the media (Fig. 2). Interaction between the bait and NubI is widely used as an external normalization factor to evaluate the relative interaction potential^28^. In contrast to this approach, we here relate the observed interaction to the NubI signal in presence of rapamycin, in order to estimate the interaction potential. While the activation of the UGT74B1 bait in combination with the SOT12-FRB-NubG prey (normalized to NubI) was indistinguishable from the negative control, the combination with SOT16 amounted some 45% of the relative interaction potential (Fig. 2), suggesting that the UGT74B1 and SOT16 interaction is of a weak nature, and highlights that the CID reporters can be employed in such cases. Under no circumstances did the presence of the stringency-increasing chemical 3-amino-1,2,4-triazole (3AT) interfere with the rapamycin-induced interactions (supplementary Fig. S3).

### CID is functional in plants and can be employed in BiFC-based analyses

**Figure 3:**
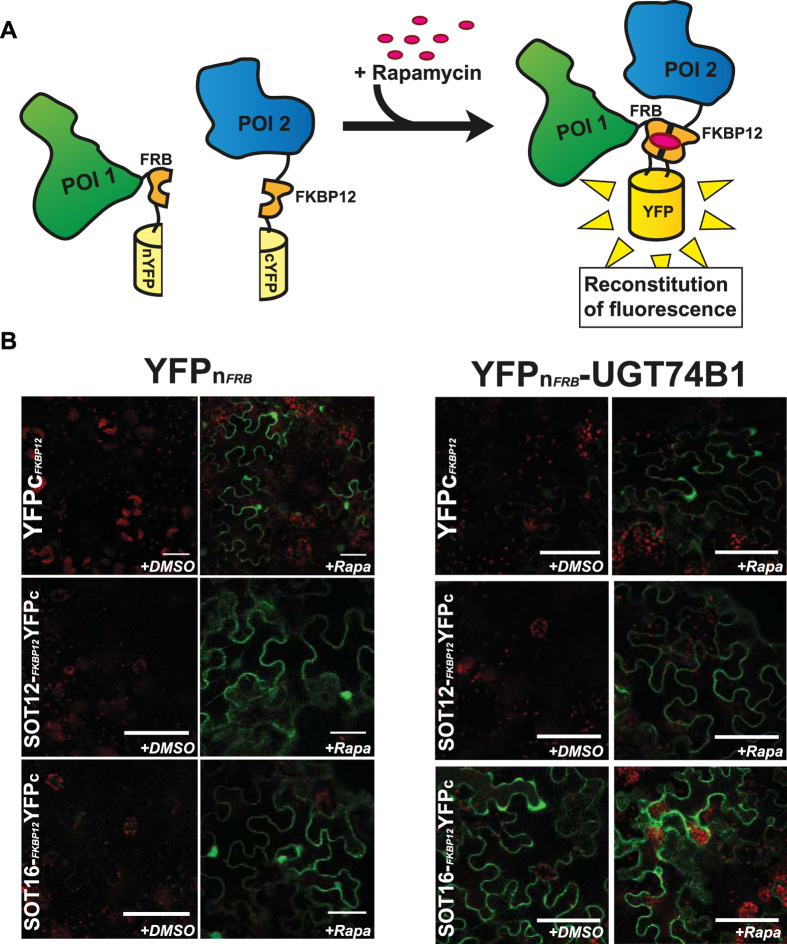
Bimolecular fluorescence complementation (BiFC) analysis of inducible interactions in Nicotiana benthamiana leaves. (**A**) Upon a negative readout indicating no interaction between the investigated proteins of interest (POI1 and POI2), the addition of rapamycin to the system will lead to induced interaction between FKBP12 and the FRB domain, and thus reassembly of the split fluorophore. (**B**) Investigation of interactions of either YFPn-FRB or YFPn-FRB-UGT74B1 with YFPc-FKBP12, SOT12-FKBP12-YFPc or SOT16-FKBP12-YFPc. The leaves were either infiltrated with water containing the carrier (+DMSO) or 30 μM rapamycin (+Rapa). The right panels each show that both partners are expressed. Scale bars represent 50 μm.

In plants, PPIs are often studied using BiFC in tobacco (*Nicotiana benthamiana*) leaves. This is achieved through infiltration of *Agrobacterium tumefaciens* bacteria into the apoplast through the abaxial leaf surface. The bacteria carry expression vectors coding for proteins fused to either the N- (YFPn) or C- (YFPc) terminal part of a fluorescent protein, thus giving rise to transient expression of the protein fusions in the transformed cells. Upon PPI, the two halves of the fluorescent protein reconstitute a functional fluorophore, which leads to fluorescence as a qualitative readout of the occurrence of PPI. Therefore, we incorporated FRB domain and FKBP12 into the YFPn and YFPc domains respectively in order to promote reconstitution in the case of a negative result (Fig. 3A).

To investigate the functionality of this CID system, we expressed the respective FKBP12- and FRB-containing YFP halves alone and in combinations containing either UGT74B1 and SOT12 or SOT16. 24 hours after infiltration with a mock solution, the YFP fluorescence found in leaves transformed with the two modified reporter tags alone was negligible under the applied conditions. In similarly treated and analysed leaves infiltrated with rapamycin, we detected a strongly increased YFP-specific fluorescence (Fig. 3B). In leaves transformed with YFPn-FRB-UGT74B1 and YFPc-FKBP12- SOT12 constructs, we observed a similar behaviour as for the empty reporter, namely an increased fluorescent signal in leaves infiltrated with rapamycin (Fig. 3B, supplementary Fig. S4). Upon treatment with the mock solution, we only detected positive signals in combinations carrying UGT74B1- and SOT16-containing fusions, indicating that these proteins also interact *in planta*. In all cases, we obtained similar results when the proteins of interest were N-terminally tagged with the reporters (supplementary Fig. S4). Taken together, these results highlight that the CID system is functional in plants and, when applied to BiFC, works qualitatively in a similar manner as for the split ubiquitin system in yeast.

**Figure 4:**
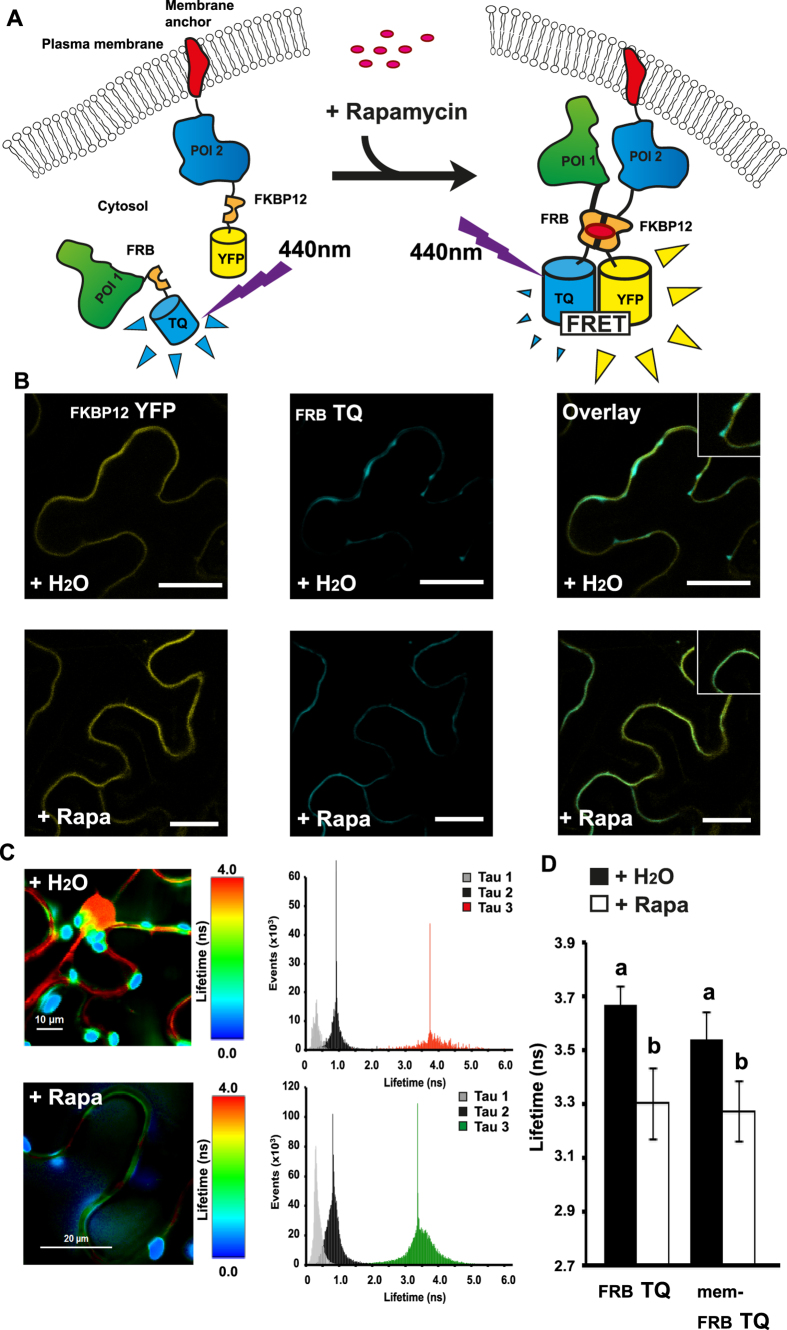
Förster Resonance Energy Transfer (FRET) analysis of inducible re-localization and interactions in Nicotiana benthamiana epidermal cells. (**A**) Upon negative interaction between two proteins of interest (POI1 and POI2), addition of rapamycin to the system will lead to re-localization of POI1 to the plasma membrane and induction of FRET. (**B**) Fluorescence signals from an epidermal cell expressing FKBP12 fused to a yellow fluorescent protein anchored to the plasma membrane (FKBP12-YFP) and FRB fused to a cyan fluorescent protein (FRB-TQ) infiltrated with either water (+H_2_O) or 30 μM rapamycin (+Rapa). The scale bars represent 50 μm. (**C**) Lifetime of the TQ signal from an epidermal cell expressing FKBP12-YFP and FRB-TQ and infiltrated with either water (+H_2_O) or 30 μM rapamycin (+Rapa). Three distinct lifetimes were resolved (Tau1-3) where Tau3 represent the signal specific to mTQ. (**D**) Average lifetime (ns) of the fluorescence from either FRB-TQ or a membrane-anchored version (mem-FRB-TQ) in combination with a membrane anchored version of FKBP12-YFP. Different letters indicate significant differences between groups (One-way ANOVA, P < 0.01, N_ _= 8–10).

Another well-established method in plant-based PPI studies is Förster Resonance Energy Transfer, (FRET)-based analysis. In this method, two proteins of interest are tagged with a donor and an acceptor fluorophore, respectively. As these are brought into close vicinity upon PPI, they undergo FRET, which is measurable as a change in characteristics of the emitted fluorescence of the donor^14^. To further demonstrate the versatility of the FKBP12- and FRB-based inducible reporter tags, we constructed expression vectors that allow fusion of proteins of interest to the CID domains followed by the fluorescent proteins mTurquoise (TQ) or mVenus (YFP) (Fig. 4A). We included a membrane anchor (mem) in the N-terminal of the YFP-containing fusions in order to evaluate chemically induced re-localization of proteins to the membrane upon rapamycin addition (Fig. 4A). As for the CID BiFC system, we employed the modified FRET system through transient expression in tobacco leaves and measured the FRET signal by fluorescence-lifetime imaging microscopy (FLIM).

Indeed, when leaves expressing the mem-FKBP12-YFP in combination with cytosolic FRB-TQ alone were infiltrated with a mock solution, we could observe areas of TQ expression in structures corresponding to the cytosol, while the YFP fluorescence appeared to outline the cell and was restricted to the membrane (Fig. 4B, insert). Upon infiltration of rapamycin, signal specific to the FRB-TQ also appeared restricted to the membrane, and overlapping with the YFP signal (Fig. 4B, insert). To further investigate if this apparent relocation to the membrane gave rise to FRET between the two fluorophores, and thus can serve as an additional validation of functionality in this system, we measured the fluorescence lifetime changes of the TQ donor. Upon infiltration with water, we observed a relatively long lifetime in areas representing the expression of the FKBP-YFP containing construct (Fig. 4C). Moreover, we observed a similar lifetime from the nucleus and transvacuolar strands, suggesting that the FKBP12-YFP construct might not be completely membrane-anchored due to overexpression. However, upon infiltration with Rapamycin, the lifetime appeared to be lower in areas representing the membrane (Fig. 4C). In both cases, we detected a strong signal with a very short lifetime from areas that correspond to chloroplasts (Fig. 4C). Upon water infiltration, the average lifetime of areas containing the membrane-anchored FKBP12-YFP, was found to be 3.67 ± 0.07 ns, whereas this decreased significantly to 3.3 ± 0.13 ns (P < 0.01) upon infiltration with rapamycin (Fig. 4D). Interestingly, when analysing the membrane-anchored FKBP12-YFP fusion in combination with a membrane anchored FRB-TQ construct, we could observe a similar rapamycin-induced decrease of the donor lifetime in areas representing the membrane (Fig. 4D) suggesting that rapamycin can also induce interactions between two membrane-anchored constructs.

### CID can be employed in FRET-based PPI analysis in plants

When investigating interaction between mem-FKBP12-YFP and SOT12 or SOT16 fused to FRB-TQ, we observed a significant rapamycin-inducible reduction of the donor lifetime upon infiltration with rapamycin for both constructs (P < 0.01) (Table 1). However, while we observed a similar decrease in lifetime in the SOT12-containing donor in combination with UGT74B1, no significant reduction was observed between UGT74B1 and SOT16 combinations (P > 0.01) (Table 1) indicating that UGT74B1 and SOT16 were also interacting in the absence of rapamycin in this system. Moreover, when calculating the changes in FRET upon presence of rapamycin (ΔFRET) we consistently observed an increase in about 10% for all combinations except UGT74B1/SOT16 where no further FRET was induced at the membrane (Table 1). This illustrates that constructs were functional in this system and capable of undergoing FRET at the membrane. In combination, these results highlight that the CID-concept is also functional for re-localization studies of proteins in tobacco epidermal cells and applicable to FLIM-based FRET analysis.

## Discussion

FKBP12 and the FRB domain originate from the TOR (Target of Rapamycin) signalling pathway, a central controller of cell growth in response to nutrient- and stress-related factors^29^. The three genes *FPR1*, *TOR1* and *TOR2* (Target of Rapamycin 1 and 2) have been identified to be responsible for rapamycin susceptibility in yeast^24^. Although a triple deletion was found to mimic the effects of rapamycin, yeasts carrying specific mutations in *TOR1* (*TOR1-1*) and deletion of *FPR1* (Δ*fpr1*) have been described as fully viable under standard conditions and have been used to perform detailed investigations of the interaction between the human FKBP12 and TOR in a yeast two-hybrid system^24^. We incorporated these mutations into the NMY51 strain carrying the genetic reporters for split-ubiquitin PPI analysis, thereby achieving rapamycin resistance up to 100 μg/mL (supplementary Fig. S1). It should be noted that the necessary modification of this central signalling pathway might be incompatible with studies analysing PPI between proteins related to the TOR pathway.

Rapamycin initially binds to FKBP12 thus creating a binding surface for FRB^30^,^31^, which upon dimerization causes a cell cycle arrest in the G1 phase^32^,^33^. Interestingly, through systematic mutation in the rapamycin binding sites of FKBP12 and FRB, artificial versions that specifically bind to analogues of rapamycin (rapalogues) have been developed^34^. By employing such combinations, it might be possible to circumvent the need to use or generate rapamycin-insensitive mutants. However, it remains to be investigated if the presence of the endogenous versions of FRB and FKBP12 gives rise to unspecific binding to the bait and prey constructs under these circumstances. Moreover, employing the easily available rapamycin and not synthetically produced rapalogues represent a more cost-effective solution.

Applications based on the inducible dimerization between FKBP12 and the FRB domains in plants are limited. Due to decreased binding of rapamycin, plants have a higher tolerance to rapamycin than other organisms^35^, although they do have a functional TOR signalling pathway^36^. However, FKBP12 from Arabidopsis and the FRB domain from humans have been found to interact in a yeast two-hybrid analysis^37^, suggesting that the introduction of FKBP12- and FRB-containing constructs might lead to increased rapamycin sensitivity. Indeed, expression of yeast FKBP12 in Arabidopsis, resulted in stunted growth and reduced protein biosynthesis^38^. The TOR pathway is mainly active in proliferative tissues and embryo development^38^,^39^. Thus, these concerns are likely of little importance in transiently transformed and fully developed leaves. In line with this, we observed a clear rapamycin-dependent induction of interaction between FKBP12 and the FRB domain (Figs 3 and 4) with no observed necrosis or phenotypic effects on the leaf. More studies are required to evaluate if FKBP12/FRB-based systems are functional in other plant tissues and/or if they interact with any endogenous proteins that might interfere with the PPI analysis under specific circumstances.

In classical split-based PPI analysis, expression of the prey construct should be confirmed by Western blotting when no interaction can be detected. However, validation of protein expression does not address the functionality of the reporter fusion and is not suitable for high-throughput analyses. In our CID split ubiquitin system, we observed that rapamycin induces interaction between the FKBP12- and FRB-containing reporters independently of the employed proteins (Figs 1 and 2). Hence, this system serves as a tool to improve the certainty that employed protein fusions are functional and non-interacting combinations indeed represent true-negatives. In order to address the relative interaction strengths, the reporter readout can be normalized to that of the positive NubI control. Yet, the lack of an internal control makes the normalization dependent on the activation level of NubI, and is thus not directly comparable between experiments as it might fluctuate from one experiment to another. This gives rise to increased variation and renders the system less sensitive. Normalization of the reporter signal to the rapamycin-induced interaction rather than directly to NubI, indicated a waek interaction between UGT74B1 and SOT16 relative to the induced internal positive control (Fig. 2). This suggests that CID might provide a suitable normalization factor to evaluate the interaction potential and address relative interaction strengths. However, further analyses using protein pairs with known binding characteristics are necessary to fully evaluate the potential of the system to detect relative interaction strengths. Split ubiquitin and yeast two-hybrid analyses can be applied to investigate interactions across entire genomes through screening of interactions between specific baits and a library of prey constructs^14^,^15^,^16^. In classical approaches, the coverage of a screen is evaluated as a ratio between the estimated individual clones in the library and amount of colony forming units (CFU) upon growth on media that is non-selective for PPI. Employing the rapamycin-inducible constructs to large-scale PPI analysis would allow for an additional estimation of the functional clones within the library and thus the proportion of true-negative interactions.

Within BiFC-based analysis of PPI in tobacco leaves, co-expression of both investigated constructs is required to occur in not only the same cell, but also the same subcellular compartment in order to achieve activation of the reporters. Thus, evaluation of a true-negative interaction is troublesome and requires the employment of a number of control experiments for correct interpretation^40^. Examples to overcome these issues include co-expression of the split reporter with a full fluorophore that has distinguishable fluorescence characteristics as well as same vector expression of both halves of the reporters^41^,^42^,^43^. By incorporating FKBP12 and the FRB domain to the C- and N-terminus of YFP we were able to address the co-expression of the reporters directly, either alone or in combination with investigated proteins (Fig. 3B). In a similar manner as for the split ubiquitin system, CID represents a built-in control that allows a direct readout of the functionality. With the ability to induce interaction between two proteins of interest, the CID concept might be employed to investigate the subcellular localization and topology of any given protein, based on the ability to achieve fluorescence upon rapamycin with N- or C-tagged versions of any two proteins expressed in different compartments. Additionally, different fluorophore fragments have been employed in combination to achieve distinctive emission spectra of specific interactions^44^. By employing the rapamycin-inducible system in such a case, it comes within reach to investigate the occurrence of specific interactions between several proteins at the same time. In contrast to the split ubiquitin and BiFC systems, in the FRET-based analysis each protein partner is tagged with a fluorescent tag that allows easy confirmation of expression. However, the ability to induce the constructs to interact through addition of rapamycin also here represents a built-in positive control as intracellular re-localization of a cytosolic protein can validate functionality of the CID when the partner is membrane-bound.

In conclusion, we have shown that implementation of the FKBP12 and FRB domains can be easily implemented into split reporter systems for PPI analysis. We show that the CID based on these small proteins is functional and can be used to induce PPI as well as subcellular re-localization in tobacco epidermal cells. Moreover, through analysis of proteins involved in GLS biosynthesis, our data demonstrate that the CID systems allow quicker establishment of interaction between proteins of interest and increased sensitivity. In combination, these improvements might help in the investigations of weak and dynamic PPIs that are likely to be an overlooked, yet important part of subcellular orchestration of biosynthetic pathways. Interrogation of PPIs in a multi-enzymatic pathway requires a variety of complementary methods dependent on the nature of the investigated proteins. Thus, CID split reporter systems represent an increased level of control that can easily be implemented into schemes aimed at PPI analysis.

## Methods

### Construction of the pFKBP12 and pFRB-N vectors and cloning of bait and prey constructs

The pFKBP12 bait vector was derived from the pDHB1 vector (Dualsystems Biotech). Cub was amplified from the original pDHB1 plasmid. FKBP12 was kindly provided by T. Gadella, (Amsterdam, NL) and the two fragments were subcloned by USER™ cloning/USER™ fusion45. The fragment containing the FKBP12-Cub fusion was ligated to NcoI and NotI digested pDHB1 vector backbone. For the insertion of bait sequences, the UGT74B1 coding sequence was amplified from Arabidopsis thaliana, Col–0 cDNA. The LargeT fragment was excised from the original pDHB1-LargeT vector (Dualsystems Biotech). Both bait sequences were inserted into the pFKBP12 vector using the SfiI restriction endonuclease (New England Biolabs) and the respective restriction sites. Likewise, the UGT74B1 sequence was inserted into the SfiI opened pDHB1 vector.

The pFRB-N prey vector was derived from the pPR3-N vector (Dualsystems Biotech). NubG was amplified from the original pPR3-N plasmid as template. The FRB fragment was kindly provided by T. Gadella (Amsterdam, NL) and the two fragments were inserted into a subcloning vector by USER™ cloning/USER™ fusion^45^. The NubG-FRB fusion sequence was inserted into a pPR3-N vector backbone using NdeI and BamHI (New England Biolabs). SOT12, SOT16 and ΔP53 cDNA sequences were amplified from *Arabidopsis thaliana*, Col–0 cDNA or the pDSL–ΔP53 vector (Dualsystems Biotech) respectively. The PCR products were inserted into SfiI sites of the pFRB-N vector. The ΔP53 fragment was additionally inserted into the pPR3-N vector. To introduce a stop codon, the oligo pair Sfi-stop-F/Sfi-stop-R was inserted into the opened vectors. Oligonucleotide sequences are listed in Supplementary Table S1.

### Generation of constructs for fluorescence microscopy

To generate the vector system for BiFC analysis we fused fragments of YFP coding for the N- and C-terminal YFP or mTQ to a USER cassette^45^ (see Supplementary Table S1 for details). In brief, the PCR products were USER cloned into the pCambia 3300 vector to generate USER compatible vectors allowing expression under control by the UBQ10 promoter^46^. All vectors were confirmed by sequencing and used as expression constructs for genes of interest by USER cloning using the Pfu-X7 polymerase^47^. A complete list of primers used and constructs generated can be found in Supplementary Table S1. All sequences were confirmed by DNA sequencing.

### Tobacco expression

For expression in *Nicotiana benthamiana*, the *Agrobacterium tumefaciens* strain GV3101 was infiltrated into the abaxial air space of 2–4-week-old plants. Co-infiltration of Agrobacterium strains containing the relevant constructs was carried out at OD_600_ = 0.01 per construct. For BiFC experiments epidermal cell layers were assayed for fluorescence 1–2 day after infiltration, while for FLIM analyses, the leaves were analysed 3–4 days after infiltration. For rapamycin-induced analysis, water containing 30 μM rapamycin (solubilized in DMSO) or DMSO was infiltrated in the abaxial airspace 24 hours before measurements.

### Yeast two-hybrid assay

The NMY51 *TOR1-1* Δ*fpr1* yeast strain was generated as previously described^23^. For expression of bait and prey combinations of interest, NMY51 *TOR1-1* Δ*fpr1* was transformed according to DUALhunter^TM^ kit instructions (Dualsystems biotech.). Competent cells were obtained by diluting O/N YPAD grown culture to OD_600_ = 0.2 followed by incubation at 30 °C (120 rpm) for 4–6 hours in YPAD. Cells were harvested (700 × *g* for 5 min), followed by brief washing in 0.1 M LiOAc (Sigma) and resuspension in 100 μL sterile water per transformation. Mastermix consisting of 600 ng DNA per plasmid (1200 ng total), 240 μL 50% PEG (Sigma), 36 μL 1M LiOAc (Sigma) and 25 μl preboiled ssDNA (2 mg/mL, Sigma) was added, followed by incubation in 42 °C water (45 min). Transformed cells were harvested by centrifugation (700 × *g* for 5 min), resuspended in 100 μL sterile H_2_O and grown at 30 °C for 3 days on synthetic complete dropout media (Sunrise Science Products) with yeast nitrogen base (Duchefa) and glucose, but lacking leucine and tryptophan (SD-LW).

### Drop test

Yeast cells were co-transformed with bait and prey plasmids by the LiAc/PEG method^48^ and selected for the presence of both plasmids on synthetic complete dropout medium (Sunrise Science Products) with yeast nitrogen base (Duchefa) and glucose, but lacking leucine and tryptophan (SD-LW). Cells from three individual colonies were grown overnight in liquid SD-LW medium, pelleted and resuspended in sterile water and brought to an OD_600_ of 0.5. Dilution series of 10^−1^ to 10^−4^ were prepared in sterile water and 5 μL of each dilution was dropped on plates containing SD-LW medium or the same medium lacking adenine and histidine (SD-AHLW). Both media were supplemented with either 10 μg/mL Rapamycin or 10 μL/mL DMSO as a solvent control. The SD-AHLW medium furthermore contained 1 mM 3-amino-1,2,4-triazole (3AT, Sigma) for the LargeT bait or 2.5 mM 3AT for the UGT74B1 bait. The plates were incubated for three days at 30 °C before analysing yeast growth.

### β-galactosidase assay

For measuring β-galactosidase activity, cells from eight individual colonies per interaction pair were grown overnight in liquid SD-LW medium supplemented with either 10 μg/mL rapamycin or 10 μL/mL DMSO and diluted to an OD_600_ of 0.5. Cells from 1 mL of suspension were spun down and lysed by two freeze-thaw cycles in liquid nitrogen/RT. The resulting lysate was resuspended in 100 μL X-gal buffer (phosphate buffered saline, pH 7.4, 0.5% (w/v) 5-Bromo-4-Chloro-3-Indolyl-β-D-Galactopyranoside (X-gal, Duchefa)) and β-galactosidase activity was determined by measuring blue coloration through absorption at 615 nm in a spectrophotometer after 2 h (UGT74B1 bait) or 17 h (LargeT bait).

### Localization analysis

All localization analyses were performed using a Leica SP5-X confocal microscope equipped with a HCX lambda blue PL APO 20 × 0.7 NA objective. Yellow fluorescent protein (YFP) was excited at 514 nm. Fluorescence was collected at 525–540 nm. mTurquoise (TQ) was excited at 405 nm or 440 nm and emission was collected at 450–500 nm. Separation of YFP and TQ signals was achieved using sequential scanning mode.

### FLIM analysis

FLIM was performed by time-correlated single-photon counting (TCSPC). Measurements were done on either a Leica SP5-X two-photon system equipped with a 63 × 1.2 NA immersion objective and a time-correlated single-photon counting (TCSPC) module (PicoHarp 300, PicoQuant, Berlin, Germany) and a Mai-Tai Ti-Sapphire laser (at 960 nm), or a PicoQuant MicroTime 200 time-resolved confocal fluorescence microscope based on an OlympusIX71 optical system, equipped with a UPLSAPO 60x PlanApochromat 1.2 NA water immersion objective and laser excitation at 440 nm. The TQ lifetime was recorded with the emission filter: 450–500 nm and detected by a FLIM PMT. The laser power was reduced so the counting was kept under 800 kcounts per second to avoid build-up effects. The samples were scanned continuously for 1 to 5 min to obtain appropriate photon numbers for reliable statistics for the fluorescence decays and the quality of the fit was judged on the basis of the chi-squared statistic, χ2, and reduced randomness of residuals. Data was analysed using Symphotime 64 (PicoQuant), with a tailfit method to recover the lifetimes from the fluorescence decays. Measurements were done on ROIs containing the membranes with a threshold setup to 50 counts and with an average of two pixels. For each condition, average values of all images recorded were used to calculate FRET efficiency. FRET was calculated using the equation: E = 1−(T_DA_/T_D_) where DA equaled the presence of rapamycin and D the absence.

### Statistical analysis

For all statistical analysis, we used R version 3.0.1 (2013-05-16)^49^. Significance between groups was tested using the lm and ANOVA function with specific differences tested post-hoc using the pairwise t.test function for multiple testing. Summary statistics was calculated using the SummaryBy from the doBy package^50^.

## Additional Information

**How to cite this article**: Andersen, T. G. *et al*. Improving analytical methods for protein-protein interaction through implementation of chemically inducible dimerization. *Sci. Rep*. **6**, 27766; doi: 10.1038/srep27766 (2016).

## Supplementary Material

Supplementary Information

## Figures and Tables

**Table 1:** Analysis of fluorescence lifetime of mTurquoise (TQ)-containing fusions in tobacco (Nicotiana benthamiana) epidermis infiltrated with either water containing solvent (+DMSO) or 30 μM rapamycin (+Rapamycin). ΔFRET depicts the increase in FRET upon infiltration with rapamycin.

fkbp12 YFP						
	+*DMSO*		+*Rapamycin*		ΔFRET	
	Lifetime (ns)	SD	Lifetime (ns)	SD	Δ%	SD
frbTQ	3.510^a^	±0.099	3.167^b^	±0.071	9.782	±2.015
SOT12–frbTQ	3.463^a^	±0.092	3.038^b^	±0.106	12.274	±3.063
SOT16–frbTQ	3.522^a^	±0.067	3.200^b^	±0.100	11.379	±3.741
**UGT74B1-fkbp12 YFP**						
	**+*****DMSO***		**+*****Rapamycin***		**ΔFRET**	
	**Lifetime (ns)**	**SD**	**Lifetime (ns)**	**SD**	**Δ%**	**SD**
frbTQ	3.667^a^	±0.071	3.300^b^	±0.131	10.000	±3.340
SOT12–frbTQ	3.567^a^	±0.112	3.188^b^	±0.083	11.081	±2.119
SOT16–frbTQ	3.313^b^	±0.099	3.144^b^	±0.053	1.562	±1.670

SD, standard deviation, individual groups (indicated by different letters, P <  0.01) were determined by one-way ANOVA and Holm-Sidak posthoc test (N = 8–10).
