# Supplementary material for: Improving analytical methods for protein-protein interaction through implementation of chemically inducible dimerization
Source: Sci Rep. 2016 Jun 10;6:27766. doi: 10.1038/srep27766 (PMC4901268; doi:10.1038/srep27766)
Supplement: Supplementary Information [file srep27766-s1.pdf]

Supplementary material

Improving analytical methods for protein-protein interaction through implementation of chemically inducible dimerization

Authors and affiliations

Tonni Grube Andersen[1,*,‡], Sebastian J. Nintemann[1], Magdalena Marek[2,#], Barbara A. Halkier[1], Alexander Schulz[1] and Meike Burow[1]

[1]Center for Dynamic Molecular Interactions (DynaMo), Department of Plant and Environmental Sciences, University of Copenhagen, Thorvaldsensvej 40, 1871 Frederiksberg C, Denmark

[2]Department of Plant and Environmental Sciences, Faculty of Science, University of Copenhagen, Thorvaldsensvej 40, 1871 Frederiksberg C, Denmark

[*]Current address: Department of Plant Molecular Biology, University of Lausanne, Biophore building, 1015 Lausanne, Switzerland

[#]Current address: Department of fundamental Microbiology, University of Lausanne, Biophore building, 1015 Lausanne, Switzerland

[‡]To whom correspondence should be addressed: tonnigrube.andersen@unil.ch

Supplementary figure 1

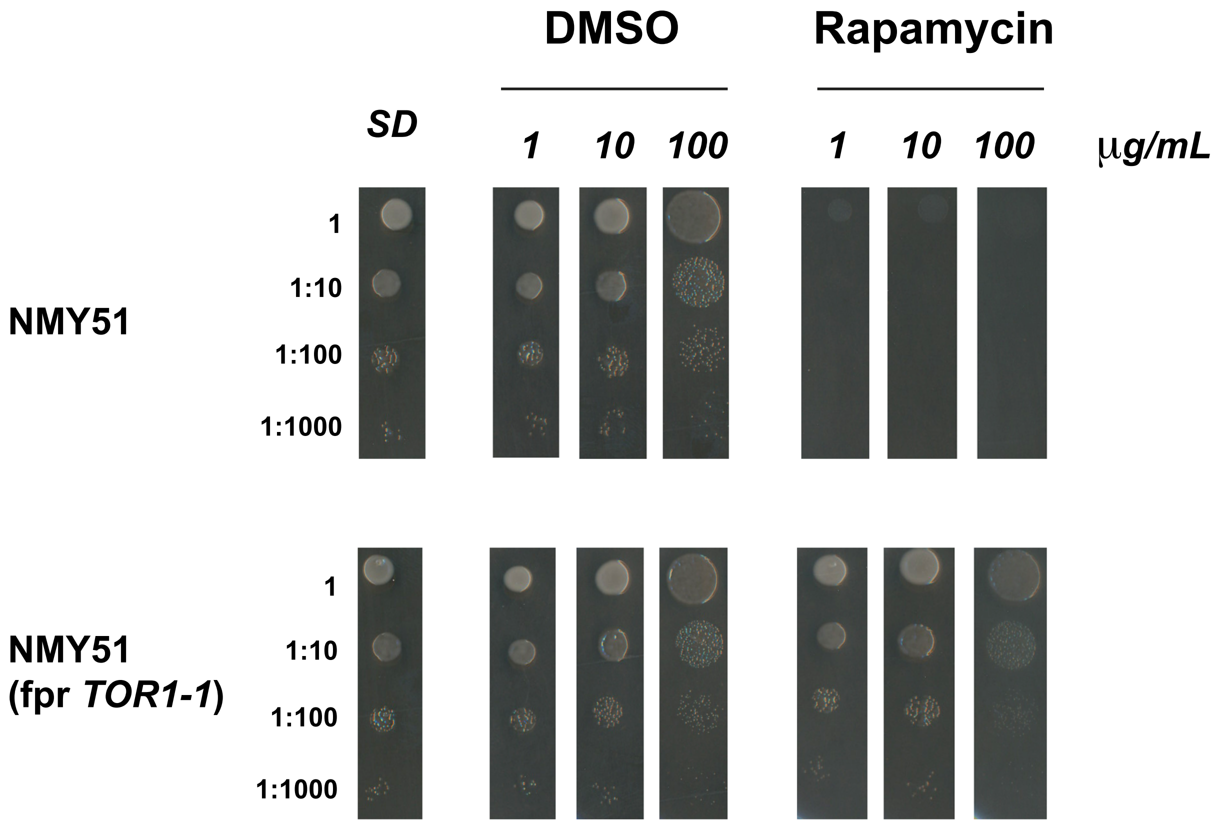

Supplementary Figure 1 Generation of NMY51 Δfpr TOR1-1 yeast line
The original NMY51 strain as well as the version containing the Δfpr and TOR1-1 mutations were evaluated on media containing a concentration gradient of either DMSO or rapamycin dissolved in DMSO.

Supplementary figure 2

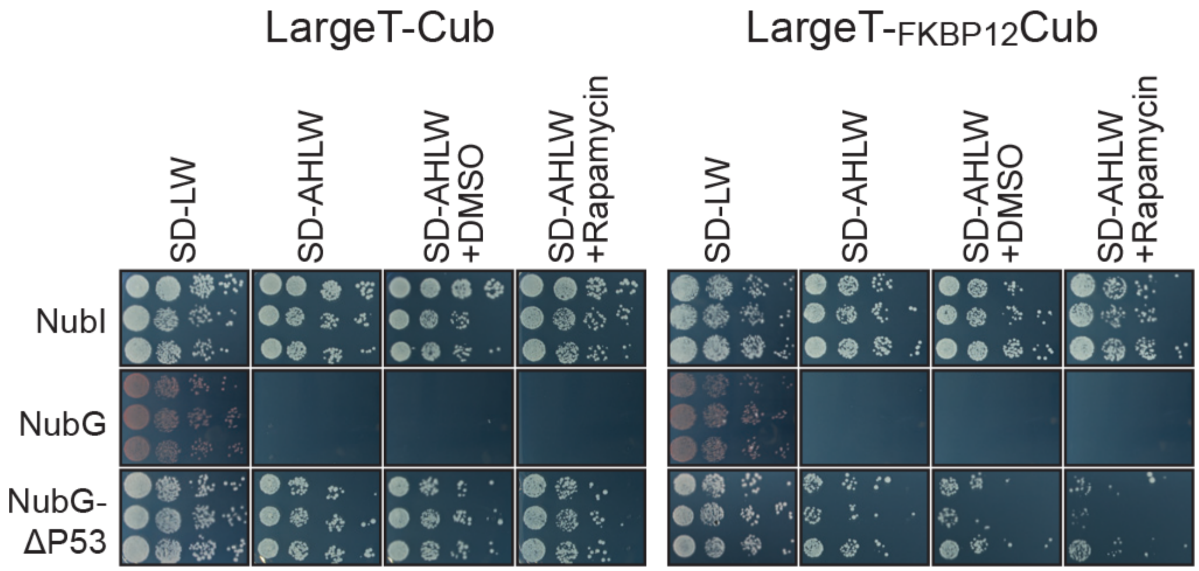

Supplementary Figure 2 yeast reporters alone and under different conditions
Growth of the rapamycin resistant yeast strain expressing LargeT bait construct with or without incorporated FKPB12 was tested against a positive control (NubI) or preys without the FRB domain present.

Supplementary figure 3

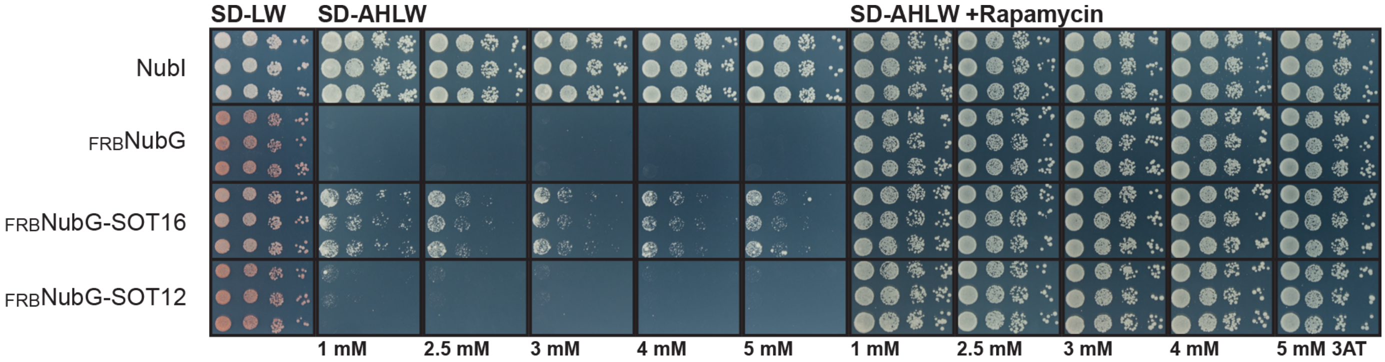

Supplementary Figure 3 UGT SOT different conditions
The interaction between the FKBP12-UGT74B1 bait and different preys was assessed under different conditions.

Supplementary figure 4

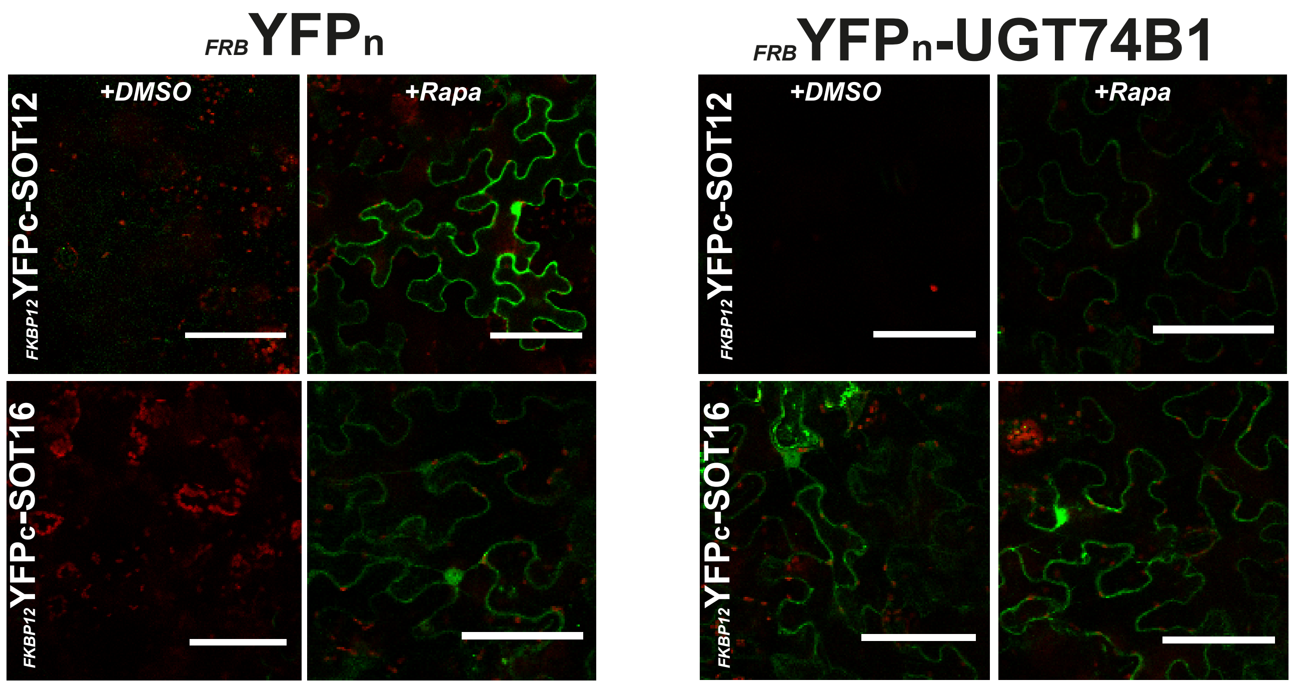

Supplementary Figure 4 N-tagged BiFC analysis
Investigation of interactions of either FRB-YFPn or FRB-YFPn-UGT74B1 against FKBP12-YFPc or the N-terminally tagged proteins FKBP12-YFPc-SOT12 or FKBP12-YF-Pc-SOT16. Tobacco leaves were either infiltrated with water containing DMSO (+ DMSO) or 30 μM rapamycin (+ Rapa). Scale bars represent 50 μm

Supplementary table 1

Construction of tobacco vectors

Number	Name	Sequence (5' to 3')
1	USER-FP forward	GGCTTAAUATGGTGAGCAAGGGCGAGGAGC
2	L1 FP R	ACCAGCACCUGAATTCGCAGATCTCTTGTACAGCTCGTCCATG
3	L1 FRB F	AGGTGCTGGUGCTGGTGCTGGTGCTATCCTATCTAGAATCCTCTGGCATGAGATGTG
4	USER- L2 R	GGTTTAAUGCTGAGGTTTAATTAAGCCTCTCAGCGGTGGCGACCGGTGCG
5	USER L1 FRB F	GGCTTAAUGCTGAGAGGCTTAATTAAACCTCAGCGCTCGAGGCGCAGCAGGGCGGGGAGTGCAGGTGGAAACC
6	FP R	GGTTTAAUTCAGTACAGCTCGTCCATGCCGAGAGTGATCCCG
7	USER L3 FKBP12 R	GGTTTAAUGCTGAGGTTTAATTAAGCCTCTCAGCCCGGTACCCTGC
8	USER L3 F	GGCTTAAUGCTGAGAGGCTTAATTAAACCTCAGCGCTCGAGGCGCAGCAGGGCGGGGAGTGCAGGTGGAAACCATCTC
9	FP FKBP12 R	AGAAGCUCCACATCGAAGACGAGAGTGGCATGTGGTGGG
10	FKBP FP F	AGCTTCUAAAACTGGAAATGGTGAGCAAGGGCGAGG
11	Yc R	GGTTTAAUCTTGTACAGCTCGTCCATGCC
12	YC F	GGCTTAAUCAGCTCGCCGACCACTACCAGCAGA ACACC
13	FRB-Yn R	ACCAGCACCUGAACAGCTCCTCGCCCTTGCTCACcat
14	FKBP YC F	AGCTTCUCAGCTCGCCGACCACTACCAGCAGA ACACC
15	Yn-FRB R	AGAAGCUGAATTCGCAGATCTCTTGTACAGCTCGTCCATG
16	FRB-Yn F	AGGTGCTGGUATGGTGAGCAAGGGCGAGGAGC
17	Yn R	GGTTTAAUGAACAGCTCCTCGCCCTTGCTCACcat

Construction of yeast vectors

Number	Name	sequence
18	USER-NcoI-Lnk-FKBI	ACCACCGCCACCUTCCAGTTTTAGAAGCTCCACATCG
19	FKBP12-FUS-R	AGGTGGCGGTGGUTCTGGTGGCGGTGGTTCTATGTCGGGGGGGATCCCTCC
20	FUS-Cub-F	GGTTTAAUTCATGCGGCCGCAAGCTGATC
21	USER-Cub-NotI-R	GGCTTAAUATGATCATATGGCATGCATGTGC
22	USER-NdeI-NubG-F	ACCGCCACCAGCGUAATCTGGAACATCGTATGG
23	FUS-NubG-R	ACGCTGGTGGCGGUGGTTCTGGTGGCGGTGGTTCTATCCTCTGGCATGAGATGTGGCATG
24	FUS-Lnk-FRB-F	GGTTTAAUGGATCCGGTGGCGACCGGTGCGTAGTC

Cloning of Genes

Number	Name	sequence
	**UGT74B1 (At1g24100)**	
25	user ugt f	GGCTTAAU ATGGCGGAAA CAACTCCCAA AGTG
26	UGT74B1-Sfi-F	ATTGAGGCCATTACGGCCATGGCGGAAACAACTCCC
27	user ugt r	GGCTTAAU TTACTTCCCTAAACTCTCTATAAACTCG
28	user ugt ns r	GGCTTAAU CTTCCCTAAACTCTCTATAAACTCG
29	UGT74B1-Sfi-R	TCAATGGCCGAGGCGGCCTTCTTCCCTAAACTCTC
	**SOT12 (At2g03760)**	
30	user 12 f	GGCTTAAU TGTCATCAT CATCATCAGT TCCTGC
31	SOT12-Sfi-F	ATTGAGGCCATTACGGCCATGTCATCATCATCATCAGTTCCTGC
32	user 12 r	GGCTTAAU TCAAGAAGAAAATTTAAGACCAGAACC
33	user 12 ns r	GGCTTAAUAGAAGAAAATTTAAGACCAGAACC
34	SOT12-Sfi-R-stop	TCAATGGCCGAGGCGGCCTCAAGAAGAAAATTTAAGACCAGAACC
	**SOT16 (At1g74100)**	
35	user 16 f	GGCTTAAUATGGAATCAA AGACAACCCA AAACGG
36	SOT16-Sfi-F	ATTGAGGCCATTACGGCCATGGAATCAAAGACAACCC
37	user 16 r	GGCTTAAU TCAGTTATCATGTTGAAGCAAGCC
38	user 16ns r	GGCTTAAUGTTATCATGTTGAAGCAAGCC
39	SOT16-Sfi-R-stop	TCAATGGCCGAGGCGGCCTCAGTTATCATGTTGAAGCAAGC
	**ΔP53**	
40	dP53-Sfi-F-start	AGAGTGGCCATTACGGCCATGG
41	dP53-Sfi-R-stop	CGAGAGGCCGAGGCGGCCTCAGTCTGAGTCAGGCC
	**Large T**	
42	Sfi-stop-F	CGGCCTAATAGCTAGGCCGCCT
43	Sfi-stop-R	CGGCCTAGCTATTAGGCCGTAA

		Cloned into USER casette of pCambia 1300	
1+2 (A)	FP	FRET vectors	
3+4 (B)	FRB-USER	A+B	TQ-FRB-USER
5+6 C	USER - FRB - TQ	C	USER -FRB -TQ
1+7 (D)	Venus FKBP12 - USER	D	Venus-FKBP12 - USER
8 + 9 (E)	USER - FKBP12	E+F	USER - FKBP12-Venus
10 +6 (F)	FKBP12 - Venus	BiFC vectors	
1+13 (G)	Yn	G+H	Yn FRB - USER
3+4 (H)	FRB - USER	I +J	USER - FRB Yn
5+15 (I)	USER - FRB	K	Yc FKBP12 - USER
16+17 (J)	Yn	L+M	USER - FKBP12 Yc
12+7 (K)	Yc FKBP12 - USER		
8+9 (L)	USER - FKBP12		
14+11 (M)	Yc		
